# Surgical specialty and preoperative medical consultation based on commercial health insurance claims

**DOI:** 10.1186/s13741-018-0089-4

**Published:** 2018-05-04

**Authors:** Stephan R. Thilen, Alex L. Woersching, Anda M. Cornea, Elliott Lowy, Edward M. Weaver, Miriam M. Treggiari

**Affiliations:** 1 Department of Anesthesiology & Pain Medicine, University of Washington, 325 Ninth Ave, Box 359724, Seattle, WA 98104 USA; 2 Department of Health Services, University of Washington, Seattle, WA 98195 USA; 3 Swedish Medical Center, Seattle, WA 98122 USA; 4 VA Puget Sound Health Care System, Seattle, WA 98108 USA; 5 Department of Otolaryngology-Head and Neck Surgery, University of Washington, Seattle, WA 98195 USA; 6 Department of Anesthesiology and Perioperative Medicine, Oregon Health & Science University, Portland, OR 97239 USA

## Abstract

**Background:**

Surgical patients are sometimes referred for preoperative evaluations by consultants in other medical specialties, although consultations are unnecessary for many patients, particularly for healthy patients undergoing low-risk surgeries. Surgical specialty has been shown to predict usage of preoperative consultations. However, evidence is generally limited regarding factors associated with preoperative consultations. This study evaluates surgical specialty and other predictors of preoperative consultations.

**Methods:**

This retrospective cohort study analyzed surgery claims of 7400 privately insured patients in Washington, United States, from eight surgical specialties. We estimated log-Poisson generalized estimating equation models that regress whether a patient received a consultation on surgical specialty and covariates accounting for the data’s hierarchical structure with patients nesting within surgeons, and surgeons nesting within provider organizations. Covariates include age, gender, Deyo comorbidity index, surgical risk, and geographic factors.

**Results:**

Overall, 485 (6.6%) patients had a preoperative consultation. The incidence of preoperative consultation varied significantly by surgical specialty. Orthopedics, neurosurgery, and ophthalmology had 3.9 (95% CI 2.4, 6.5), 2.3 (95% CI 1.1, 4.5), and 2.3 (95% CI 1.1, 4.6) times greater adjusted likelihoods of preoperative consultation than general surgery, respectively. The adjusted likelihoods of consultation for gynecology, urology, otolaryngology, and vascular surgery were not statistically different from general surgery. The following covariates were associated with greater likelihood of preoperative consultation: greater age, higher surgical risk, having one or more comorbidities vs. none, and small rural towns vs. urban areas. More than 75% of all consultations were provided to patients with a Deyo comorbidity index of 0 or 1. Low surgical risk patients had 0.3 (95% CI 0.3, 0.5) times the likelihood of preoperative consultation of intermediate and high-risk patients overall.

**Conclusions:**

The likelihood of preoperative consultation varied fourfold (an absolute 9% points) across surgical specialties. Most consultations were provided to patients with low comorbidity and with low or intermediate surgical risk. To improve usage of preoperative consultations as an evidence-based practice, future research should determine how the health outcomes effects of preoperative consultations vary depending on comorbidity burden and surgical risk.

## Background

Prior to surgical procedures, all patients undergo preoperative and pre-anesthetic evaluations by their surgeons and anesthesia providers. In the Unites States (US), global fees for surgeons and anesthesiologists compensate for those evaluations. Surgeons and anesthesia providers refer a subset of surgical patients for preoperative evaluations by consultants in other medical specialties, which the consultant separately bills for and may initiate downstream testing and care at additional expense. Evidence is limited about the appropriateness of preoperative consultation usage and the value of the resources expended on consultations.

Previous studies have reported wide variability in usage of preoperative medical consultation (Fleisher et al. 2009; Evaluation ASoATFoP 2002), which is consistent with the absence of clear recommendations from evidence-based practice guidelines directing how to select surgical patients for referral to other specialties (Katz et al. n.d.; Clelland et al. 1996; Auerbach et al. 2007; Wijeysundera et al. 2010). Indeed, a survey of anesthesiologists and surgeons involved in referrals for preoperative consultations indicated substantial disagreement among the specialties as to the reasons for obtaining consultations (Katz et al. 1998). Evidence is also varied about the risk factors that trigger referral for preoperative consultation. Previous studies have suggested that comorbidity burden is an important determinant (Auerbach et al. 2007; Wijeysundera et al. 2010); however, an analysis of Medicare claims for patients undergoing cataract surgery found that non-medical factors primarily explained variation in usage of preoperative consultations (Thilen et al. 2014).

In an earlier analysis of an integrated health care system, surgical specialty helped predict usage of preoperative consultations (Thilen et al. 2013). The finding has not been assessed for generalizability in other settings. Therefore, we analyzed commercial insurance claims from the Northwest US to test the hypothesis that surgical specialty is an important predictor of referral for preoperative medical consultation among commercially insured patients.

## Methods

The University of Washington Human Subjects Division reviewed this study and determined that it is not a human subject research.

### Design, population, and data sources

We evaluated a retrospective cohort of surgical claims from Premera Blue Cross, a commercial insurance company based in Seattle, Washington (WA). All patients were Washington State residents, aged 18–80 years old, and had undergone one of 30 a priori selected common inpatient or outpatient surgical procedures in calendar year 2010. We selected surgical procedures to include commonly performed procedures representing a spectrum of surgical risk (see the “Appendix” section for included procedures and their frequencies in the study cohort). We included eight different surgical specialties: general surgery, ophthalmology, gynecology, orthopedics, urology, otolaryngology, neurosurgery, and vascular surgery. Surgical events were nested within surgeons, and surgeons were nested within provider organizations.

We identified eligible procedures by the patient’s first occurrence of a *Current Procedural Terminology* (CPT) code for one of the specified procedures. We identified the primary endpoint—preoperative medical consultation within the 42-day preoperative period—by the presence of any CPT code for outpatient consultation (99242-99245) or inpatient consultation (99252-99255). We included consultations provided by family physicians, general internists, pulmonologists, cardiologists, endocrinologists, or nurse practitioners. We also included office visits modified with an *International Classification of Diseases, 9th Revision, Clinical Modification (ICD-9-CM)* code v72.81-v72.84, indicating that the visit was a separately billed preoperative evaluation. We chose a period of 42 days preceding surgery to maximize the ability to capture preoperative consultations that may have led to a delay in scheduling the surgical procedure.

### Covariates

We calculated the Deyo comorbidity index using diagnostic codes present within 365 days before surgery (Deyo et al. 1992). The Deyo comorbidity index was used as an ordinal variable with categories 0, 1, 2, and ≥ 3. We also calculated the Revised Cardiac Risk Index (RCRI) (Lee et al. 1999), which predicts cardiac complications. However, we did not include the RCRI in the final analyses because very few patients were class III or IV risk (98.6% were class I or II), and the RCRI index did not significantly predict consultation after adjusting for the Deyo index. Surgical procedures were categorized into low, intermediate, and high levels of surgical risk. Patient demographics included age and gender. We used the ZIP code of patient residence to allocate patients to seven hospital referral regions (HRRs) (Everett, Olympia, Seattle, Spokane, Tacoma, and Yakima (all WA cities), and Portland, OR) and to determine patient’s Rural Urban Commuting Area (RUCA) category (urban, large rural city, small rural town, and isolated rural town). HRRs are approximate health care markets for tertiary care with at least one hospital that performs major cardiovascular and neurosurgery procedures (Dartmouth Medical School and Center for the Evaluative Clinical Sciences 1996). RUCA codes are a classification scheme for characterizing all of US ZIP codes according to their urban or rural character (WWAMI Rural Health Research Center n.d.).

### Statistical analysis

We summarized counts of consultation and we ordered them by day preceding surgery up to 42 days before the index surgery. We conducted bivariate analyses to compare characteristics of patients who did or did not undergo preoperative consultation. We estimated the association between preoperative consultation (outcome variable) and surgical specialty (explanatory variable of interest) by fitting a log-Poisson generalized estimating equation (GEE) regression model with robust standard errors. GEE permits obtaining marginal population-averaged interpretations of hierarchical data using non-linear link functions. The exponents of beta coefficients from a log-Poisson model are true risk ratios in contrast to the odds ratios produced by a logistic regression. Odds ratios approximate risk ratios but are inflated (i.e., overstate effects) when outcomes are more common or as effects become larger (Kleinman and Norton 2009). Since surgeons are nested within provider organizations without any non-nested clusters, we clustered on provider organization, and the robust standard errors account for clustering at both the provider and organization levels (Betensky et al. 2000). We adjust for potential confounders: age, gender, Deyo comorbidity index, surgical risk, HRRs, and RUCA categories. The log-Poisson model passed model diagnostic tests: the Hosmer-Lemeshow statistic for model misspecification, the Pearson correlation of the raw-scale residuals and predictions, and Pregibon’s link test. A two-sided α level of 0.05 was considered for statistical significance. We report effects as relative risks in the text and Table 2 and also report selected absolute adjusted model estimates in the text and Fig. 2 (main effects model only), as specified (hereafter*, adjusted likelihoods*). All statistical analyses were performed using Stata 14 (Stata Corporation, College Station, TX, USA).

## Results

**Fig. 1:**
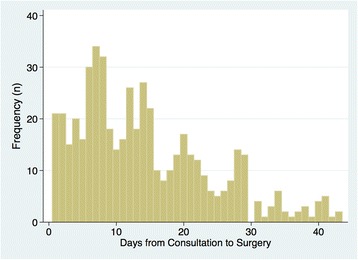
Frequency distribution of preoperative consultations in the 42 days preceding the index surgery, showing a bimodal distribution with peaks on preoperative days 7 and 14

A total of 7400 unique patients were identified undergoing any of the 30 included procedures (“Appendix” section). The characteristics of the cohort are presented in Table 1. The mean age was 50.9 years (SD 12.1), and 60% were female. Overall, 485 (6.6%) patients underwent preoperative consultation. The distribution of the timing of preoperative consultations during the days preceding surgery showed peaks on weekly intervals, with modes on days 7 and 14 (Fig. 1). The median interval from consultation to surgery was 13 days (interquartile range 7–21).

**Table 1:** Characteristics of patients receiving and not receiving preoperative consultation

	No consultation	Consultation
Total, *n* (%)	6915 (93.4)	485 (6.6)
Age, mean (SD)	50.5 (12.2)	55.5 (9.7)
Age categories, *n* (%)		
18–34	811 (97.5)	21 (2.5)
35–44	1076 (97.1)	32 (2.9)
45–54	1942 (93.6)	133 (6.4)
55–64	2542 (91.2)	246 (8.8)
65–80	544 (91.1)	53 (8.9)
Sex, *n* (%)		
Male	2743 (92.5)	224 (7.5)
Female	4172 (94.1)	261 (5.9)
Surgeon specialty, *n* (%)		
General surgery	1670 (96.9)	53 (3.1)
Gynecology	675 (98.3)	12 (1.7)
Urology	350 (92.3)	29 (7.7)
Ophthalmology	945 (96.1)	38 (3.9)
Orthopedics	2654 (89.3)	317 (10.7)
Neurosurgery	113 (89.0)	14 (11.0)
Otolaryngology	448 (96.8)	15 (3.2)
Vascular	60 (89.6)	7 (10.4)
Surgical risk, *n* (%)		
Low	3928 (95.6)	179 (4.4)
Intermediate	2284 (89.7)	261 (10.3)
High	703 (94.0)	45 (6.0)
Deyo comorbidity score, *n* (%)		
0	4641 (94.4)	275 (5.6)
1	1113 (91.9)	98 (8.1)
2	704 (90.8)	71 (9.2)
3+	457 (91.8)	41 (8.2)
Rural Urban Commuting Area category, *n* (%)		
Urban	5665 (93.3)	407 (6.7)
Large rural city	589 (96.2)	23 (3.8)
Small rural town	357 (90.4)	38 (9.6)
Isolated rural town	304 (94.7)	17 (5.3)
Hospital referral region, *n* (%)		
Everett, WA	589 (93.3)	42 (6.7)
Olympia, WA	399 (98.5)	6 (1.5)
Seattle, WA	323 (97.9)	7 (2.1)
Spokane, WA	2509 (90.2)	274 (9.8)
Tacoma, WA	1923 (94.5)	111 (5.5)
Yakima, WA	781 (96.9)	25 (3.1)
Portland, OR	391 (95.1)	20 (4.9)

All variable distributions are significantly different between the consultation and no consultation groups at *p* ≤ 0.01

**Fig. 2:**
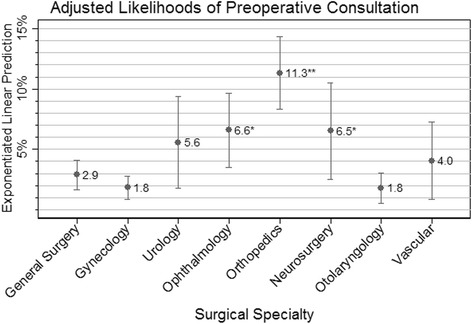
Adjusted likelihoods of preoperative medical consultation from the main effects model for each surgical specialty. Adjusted for surgical risk, age, gender, Deyo comorbidity score, urban/rural character of patient residence ZIP code, and hospital referral region. General surgery is referent, *p value = 0.02; **p value < 0.001

Table 2 shows unadjusted and adjusted relative risks of patients having a preoperative medical consultation. Figure 2 shows adjusted likelihoods of preoperative medical consultation from the main effects model for each surgical specialty. In the adjusted analysis, orthopedics, neurosurgery, and ophthalmology were associated with 3.9 (95% CI 2.4, 6.5), 2.3 (95% CI 1.1, 4.5), and 2.3 (95% CI 1.1, 4.6) times greater likelihoods of preoperative medical consultation than general surgery (referent with adjusted likelihood 2.9% (95% CI 1.7, 4.1)), respectively. Gynecology, urology, otolaryngology, and vascular surgery were not significantly different from general surgery. In the multivariate analysis, the following covariates were associated with greater likelihood of preoperative consultation: older age, higher surgical risk, comorbidities vs. none, and small rural towns vs. urban areas. Non-Seattle hospital referral regions had a 0.2–0.7 times smaller likelihood of preoperative consultation than Seattle (adjusted likelihood 8.6% (95% CI 6.5, 10.6%), except for Everett (the HRR bordering Seattle to the north) which was not significantly different from Seattle (Table 2).

**Table 2:** Crude and adjusted risk ratios for the Association of Surgical Specialty and Preoperative Consultation within 42 days

	Unadjusted risk ratios (95% confidence interval)
Surgical specialty (Ref: general surgery)	
Gynecology	0.6 (0.3, 1.2)
Urology	2.4 (0.7, 8.0)
Ophthalmology	1.8 (0.9, 3.8)
Orthopedics	*3.4 (1.9, 6.2)*
Neurosurgery	*3.4 (1.5, 7.6)*
Otolaryngology	0.8 (0.3, 2.0)
Vascular	*2.8 (1.3, 5.9)*
	Adjusted risk ratios (95% confidence interval)
Surgical specialty (Ref: general surgery)	
Gynecology	0.6 (0.3, 1.2)
Urology	1.9 (0.8, 4.9)
Ophthalmology	*2.3 (1.1, 4.6)*
Orthopedics	*3.9 (2.4, 6.5)*
Neurosurgery	*2.3 (1.1, 4.5)*
Otolaryngology	0.6 (0.3, 1.4)
Vascular	1.4 (0.7, 2.9)
Low surgical risk (Ref: intermediate and high risk)	*0.3 (0.3, 0.5)*
Age (10-year change)	*1.2 (1.1, 1.4)*
Sex (Ref: male)	
Female	1.0 (0.9, 1.2)
Deyo comorbidity score (Ref: 0)	
1	*1.4 (1.1, 1.7)*
2	*1.7 (1.3, 2.1)*
3+	*1.4 (1.1, 1.9)*
Rural Urban Commuting Area category (Ref: urban)	
Large rural city	0.8 (0.5, 1.2)
Small rural town	*1.6 (1.2, 2.1)*
Isolated rural town	0.8 (0.5, 1.3)
Hospital referral region (Ref: Seattle)	
Everett, WA	0.8 (0.5, 1.3)
Olympia, WA	*0.2 (0.1, 0.4)*
Spokane, WA	*0.7 (0.6, 1.0)*
Tacoma, WA	*0.4 (0.2, 0.7)*
Yakima, WA	*0.6 (0.4, 0.9)*
Portland, OR	*0.3 (0.2, 0.5)*

Significant findings at *p* < 0.05 are in italics

A sensitivity analysis, including only consultations within 30 days prior to surgery, reduced the number of consults by 7.4% (*n* = 36) and did not meaningfully change our results.

## Discussion

In this cohort of commercially insured patients in the state of Washington, US, we found that some surgical specialties strongly predicted referral for preoperative consultation after adjusting for age, sex, surgical risk, comorbidity burden, and geographic factors. Consistent with our previous work, which studied patients in an integrated health care system (Thilen et al. 2013), we found that ophthalmology and orthopedics were associated with greater usage of preoperative medical consultations relative to general surgery. Neurosurgery, which was not included in the previous report, was also associated with a higher usage of preoperative consultations than general surgery in the current study. Relative to general surgery, we did not find an increased likelihood of preoperative medical consultation among urology patients, although this cohort included only 379 patients. Similar to our previous report, gynecology had the lowest association with preoperative medical consultations, although not significantly different from general surgery. Gynecologists often provide general medical care (Rosenblatt et al. 1998), which supports the view that this care model may reduce usage of preoperative medical consultations for patients undergoing gynecological surgery. In our primary model, low surgical risk patients had two thirds lower likelihood of preoperative consultation than intermediate and high-risk patients.

Preoperative medical consultations were more likely for patients with some comorbidity compared to no comorbidity; however, > 75% of all consultations were provided to patients with a Deyo comorbidity index of 0 or 1. It is not clear what considerations lead to usage of preoperative medical consultations for these low-risk patients, who were often undergoing low-risk surgeries. Medical consultations do provide the opportunity to improve documentation of comorbidities, perform risk stratification, optimize factors associated with preexisting medical conditions, and initiate interventions intended to decrease perioperative risk (such as perioperative beta-blockers) (Devereaux et al. 2000; Pausjenssen et al. 2008). However, these potential objectives apply less to patients with minimal or no comorbidities, especially those undergoing low-risk surgeries.

It is possible that while all surgeons are trained to obtain a medical history and perform a complete physical examination, some specialists provide mainly a focused examination and prefer not to personally evaluate the patient for comorbidities. It is possible that some surgeons view this approach as efficient because it allows the surgeon to spend less time on the general medical evaluation and focus more time and effort on the evaluation for and performance of the surgical procedures themselves. Future studies are needed to ascertain to what extent this is an explanatory factor for referral to preoperative medical evaluation. To determine the role of preoperative medical consultations in adding value to perioperative care, it will be important to demonstrate improved outcomes such as reduced medical and surgical complications, decreased length of stay, improved recovery (e.g., for outpatient surgery, fewer postoperative emergency room visits), or better patient-centered outcomes. Presumably, such benefits would be most likely for patients with high medical and/or surgical risk. Reports have also shown a potential risk of over-diagnosis among patients referred for cardiology consultation (Sheffield et al. 2013). Future research is also needed to evaluate the cost consequences of current practice and to determine the role of preoperative consultations.

### Limitations

This study is based on private insurance claims, which may be affected by coding errors or misclassification of preoperative consultation. Consultations most commonly occurred on preoperative days 7 and 14, and with a very low occurrence of visits preceding preoperative day 30, which suggests that most of these visits were associated with a planned surgery (i.e., low misclassification of unrelated visits as preoperative consultations). Furthermore, this pattern’s consistency with prior findings (including our study of an integrated health care system) corroborates the construct validity of our measure of preoperative consultations.

If preoperative medical consultations delayed or canceled surgeries, our data would not include those consultations. However, we believe this limitation is minor, as we used a relatively long, 42-day preoperative window. It is also unknown whether the information resulting from these consultations altered the patient perioperative management. Nevertheless, we have demonstrated that there is a substantial usage of preoperative medical consultation also for patients with limited or no comorbidities, many of whom are undergoing low-risk surgeries. Although this sample is relatively large for studies of preoperative consultation in the US, the sample still had limited power to detect small effects. The study sample included a small number of vascular surgery and neurosurgery patients, and this limits our ability to draw conclusions specific to these specialties regarding consultations. In the absence of a broad consensus regarding an optimal surgical-risk categorization for all procedures listed, we arbitrarily assigned surgical risk based on similarities with other known procedures in the same categories, and based on clinical judgment. This risk categorization imperfectly differentiates average risk across procedures. We included other covariates to additionally adjust for individual- and geographic-specific factors that predict preoperative consultations, although we could not entirely eliminate selection bias in this observational study.

## Conclusions

In summary, in this study using commercial insurance claims, we found that surgical specialty is associated with the usage of preoperative medical consultation. Although surgical risk strongly predicted usage of consultations, surgical risk did not affect the likelihood of consultation within any individual surgical specialty. Our results extend our previous findings from a study in a single integrated health care system, which also demonstrated variation in preoperative consultation usage by surgical specialty, and found that most consultations were provided for patients with low medical and/or surgical risk. Given these findings, there is a pressing need for outcome studies to evaluate the impact of preoperative medical consultations and guide their optimal use.

## Appendix

**Table 3:** List of surgical procedures

Surgeon specialty and index surgery	No consultation	Had a consultation	% with a consultation
Total	6915	485	6.6
Otolaryngology	448	15	3.2
Ethmoidectomy and endoscopic sinus surgery^I^	440	15	3.3
Non-endoscopic sinus surgery^I^	8	0	0.0
General surgery	1670	53	3.1
Colectomy^H^	124	8	6.1
Exploratory laparotomy^H^	10	2	16.7
Inguinal hernia repair^L^	327	7	2.1
Laparoscopic cholecystectomy^I^	853	25	2.8
Mastectomy^L^	306	8	2.5
Node biopsy^L^	50	3	5.7
Gynecology	675	12	1.7
Dilation and curettage^L^	128	1	0.8
Hysterectomy^H^	424	11	2.5
Tubal ligation^L^	123	0	0.0
Neurosurgery	113	14	11.0
Carpal tunnel release^L^	4	1	20.0
Craniotomy^H^	39	3	7.1
Laminectomy, lumbar^I^	70	10	12.5
Ophthalmology	945	38	3.9
Cataract surgery^L^	838	30	3.5
Ptosis repair^L^	49	5	9.3
Vitrectomy^I^	58	3	4.9
Orthopedics	2654	317	10.7
Carpal tunnel release^L^	291	12	4.0
Femur fracture repair^I^	6	0	0.0
Knee arthroscopy^L^	1595	105	6.2
Laminectomy, lumbar^I^	123	17	12.1
Total hip arthroplasty^I^	252	70	21.7
Total knee arthroplasty^I^	387	113	22.6
Urology	350	29	7.7
Laparoscopic radical prostatectomy^H^	60	16	21.1
Lithotripsy^L^	217	7	3.1
Radical prostatectomy^H^	29	3	9.4
Transurethral prostatectomy^I^	44	3	6.4
Vascular	60	7	10.4
AV fistula^I^	24	2	7.7
Carotid endarterectomy^I^	19	3	13.6
Endovascular AAA repair^H^	3	0	0.0
Femoro-popliteal bypass^H^	12	2	14.3
Open AAA repair^H^	2	0	0.0

^L^Low risk

^I^Intermediate risk

^H^High risk

## Abbreviations

CPT: Current Procedural Terminology
GEE: Generalized estimating equation
HRR: Hospital referral region
ICD: International Classification of Diseases
OR: Odds ratio
OR: Oregon
RCRI: Revised Cardiac Risk Index
RUCA: Rural Urban Commuting Area
US: United States
WA: Washington
